# Atomoxetine restores the response inhibition network in Parkinson’s disease

**DOI:** 10.1093/brain/aww138

**Published:** 2016-06-24

**Authors:** Charlotte L. Rae, Cristina Nombela, Patricia Vázquez Rodríguez, Zheng Ye, Laura E. Hughes, P. Simon Jones, Timothy Ham, Timothy Rittman, Ian Coyle-Gilchrist, Ralf Regenthal, Barbara J. Sahakian, Roger A. Barker, Trevor W. Robbins, James B. Rowe

**Affiliations:** ^1^ 1 Department of Clinical Neurosciences, University of Cambridge, Cambridge, CB2 0SZ, UK; ^2^ 2 Medical Research Council Cognition and Brain Sciences Unit, Cambridge, CB2 7EF, UK; ^3^ 3 Division of Clinical Pharmacology, Rudolf-Boehm-Institute of Pharmacology and Toxicology, University of Leipzig, Leipzig, 04107, Germany; ^4^ 4 Behavioural and Clinical Neuroscience Institute, Cambridge, CB2 3EB, UK; ^5^ 5 Department of Psychiatry, University of Cambridge, CB2 0SZ, Cambridge, UK; ^6^ 6 Department of Experimental Psychology, University of Cambridge, CB2 3EB, Cambridge, UK

**Keywords:** atomoxetine, effective connectivity, Parkinson’s disease, response inhibition, stop-signal task

## Abstract

Parkinson’s disease impairs the inhibition of responses, and whilst impulsivity is mild for some patients, severe impulse control disorders affect ∼10% of cases. Based on preclinical models we proposed that noradrenergic denervation contributes to the impairment of response inhibition, via changes in the prefrontal cortex and its subcortical connections. Previous work in Parkinson’s disease found that the selective noradrenaline reuptake inhibitor atomoxetine could improve response inhibition, gambling decisions and reflection impulsivity. Here we tested the hypotheses that atomoxetine can restore functional brain networks for response inhibition in Parkinson’s disease, and that both structural and functional connectivity determine the behavioural effect. In a randomized, double-blind placebo-controlled crossover study, 19 patients with mild-to-moderate idiopathic Parkinson’s disease underwent functional magnetic resonance imaging during a stop-signal task, while on their usual dopaminergic therapy. Patients received 40 mg atomoxetine or placebo, orally. This regimen anticipates that noradrenergic therapies for behavioural symptoms would be adjunctive to, not a replacement for, dopaminergic therapy. Twenty matched control participants provided normative data. Arterial spin labelling identified no significant changes in regional perfusion. We assessed functional interactions between key frontal and subcortical brain areas for response inhibition, by comparing 20 dynamic causal models of the response inhibition network, inverted to the functional magnetic resonance imaging data and compared using random effects model selection. We found that the normal interaction between pre-supplementary motor cortex and the inferior frontal gyrus was absent in Parkinson’s disease patients on placebo (despite dopaminergic therapy), but this connection was restored by atomoxetine. The behavioural change in response inhibition (improvement indicated by reduced stop-signal reaction time) following atomoxetine correlated with structural connectivity as measured by the fractional anisotropy in the white matter underlying the inferior frontal gyrus. Using multiple regression models, we examined the factors that influenced the individual differences in the response to atomoxetine: the reduction in stop-signal reaction time correlated with structural connectivity and baseline performance, while disease severity and drug plasma level predicted the change in fronto-striatal effective connectivity following atomoxetine. These results suggest that (i) atomoxetine increases sensitivity of the inferior frontal gyrus to afferent inputs from the pre-supplementary motor cortex; (ii) atomoxetine can enhance downstream modulation of frontal-subcortical connections for response inhibition; and (iii) the behavioural consequences of treatment are dependent on fronto-striatal structural connections. The individual differences in behavioural responses to atomoxetine highlight the need for patient stratification in future clinical trials of noradrenergic therapies for Parkinson’s disease.

## Teaser

Impairments in response inhibition in Parkinson’s disease may reflect loss of noradrenaline and impaired fronto-subcortical connectivity. Rae *et al*. show that the noradrenaline reuptake inhibitor atomoxetine can restore functional connectivity in the inhibition network. Individual treatment responses depend on disease severity, plasma drug concentration and anatomical connectivity within the network.

## Graphical abstract

**Figure:**
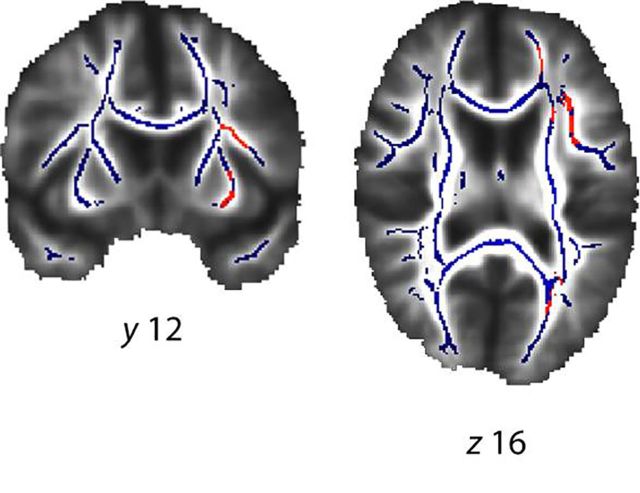
Impairments in response inhibition in Parkinson’s disease may reflect loss of noradrenaline and impaired fronto-subcortical connectivity. Rae *et al*. show that the noradrenaline reuptake inhibitor atomoxetine can restore functional connectivity in the inhibition network. Individual treatment responses depend on disease severity, plasma drug concentration and anatomical connectivity within the network.

## Introduction

Parkinson’s disease is a complex disorder, in which the cardinal features of bradykinesia, rigidity and tremor are often accompanied by cognitive changes, even at diagnosis (Nombela *et al.*, 2014*b*; Yarnall *et al.*, 2014). A dysexecutive syndrome is common, including impairment of response inhibition even in the absence of clinically severe impulse control disorders (Napier *et al.*, 2014). For example, despite bradykinesia, Parkinson’s disease impairs performance on a stop-signal task (Obeso *et al.*, 2011*a*; Nombela *et al.*, 2014*a*; Ye *et al.*, 2014). When stopping an action, patients show abnormal responses in cortical and subcortical regions, including the inferior frontal gyrus, pre-supplementary motor area (preSMA), and subthalamic nuclei (Frank *et al.*, 2007; Ballanger *et al.*, 2009).

To restore inhibitory control in Parkinson’s disease, we drew on animal models that indicate a role for noradrenaline in regulating response inhibition and impulsivity (Eagle and Baunez, 2010; Bari *et al.*, 2011; Bari and Robbins, 2013), notwithstanding the contributory role of fronto-striatal anatomical connections that are also abnormal in Parkinson’s disease (Rae *et al.*, 2012). Specifically, we proposed that the loss of noradrenergic neurons and their projections to the cortex (Goldstein *et al.*, 2011) promote impulsivity, over and above changes in dopaminergic function and dopaminergic treatment (Vazey and Aston-Jones, 2012). The potential for the selective noradrenaline reuptake inhibitor (SNRI) atomoxetine to modulate response inhibition systems is suggested by recent studies in Parkinson’s disease (Kehagia *et al.*, 2014; Ye *et al.*, 2015, 2016; Borchert *et al.*, 2016), attention deficit hyperactivity disorder (Chamberlain *et al.*, 2007; Cubillo *et al.*, 2014), and healthy subjects (Chamberlain *et al.*, 2009).

Pharmacological enhancement of regional brain activity is unlikely to alter behaviour if that region is functionally disconnected from downstream effector mechanisms. As neurotransmitters influence the communication between neuronal populations, drug effects may be understood better in terms of connectivity between regions rather than activity within regions (Rowe, 2010; Moran *et al.*, 2013). For inhibitory control, communication between the inferior frontal gyrus, preSMA and their projections to the subthalamic nucleus are of particular relevance: lesion studies, transient interference by magnetic or electrical stimulation, and neuroimaging provide convergent evidence that interactions amongst these three regions are crucial for successful response inhibition (Chambers *et al.*, 2006; Frank *et al.*, 2007; Duann *et al.*, 2009; Forstmann *et al.*, 2012; Aron *et al.*, 2014; Rae *et al.*, 2015).

We therefore used functional MRI to test the hypothesis that atomoxetine restores connectivity within the inhibitory control network in Parkinson’s disease. We used the analytic framework of Dynamic Causal Modelling (DCM, Stephan *et al.*, 2008), with evidence-based model selection procedures that are robust in the context of Parkinson’s disease (Rowe *et al.*, 2010; Herz *et al.*, 2014). Moreover, the estimated parameters of directional connectivity from one region to another correlate with neurophysiological and anatomical markers of connectivity (Boudrias *et al.*, 2012; Rae *et al.*, 2015). We sought additional evidence from the correlations between the behavioural effect of atomoxetine, functional connectivity, structural connectivity, drug levels and disease severity. Given the heterogeneity of Parkinson’s disease, we predicted that disease severity and white matter structure would influence changes in functional connectivity in the inhibitory network and response inhibition performance.

## Materials and methods

### Subjects and experimental design

Nineteen patients with idiopathic Parkinson’s disease (UK Parkinson’s Disease Society Brain Bank Clinical Diagnostic Criteria) were recruited from the PD Research Clinic at the John van Geest Centre for Brain Repair. Inclusion criteria were: (i) Hoehn and Yahr stage 1.5–3; (ii) age 50–80 years; (iii) right-handed; and (iv) non-demented, using DSM-IV criteria and Mini-Mental State Examination ≥26/30 at recent clinical assessment. Exclusion criteria were (i) clinically significant current depression; (ii) contraindications to MRI or atomoxetine; and (iii) a history of significant psychiatric disorder. Patients were tested on their normal anti-parkinsonian dopaminergic medication (‘ON’ state). Levodopa equivalent daily dose (LEDD) was calculated according to the formula of Tomlinson *et al.* (2010).

In a double-blind randomized placebo-controlled crossover design, patients participated in two separate study sessions, at least 6 days apart. The design is similar to that reported by Ye *et al.* (2015) in a separate group of patients. Patients received either 40 mg of oral atomoxetine or placebo. Blood samples were collected 2 h after drug administration, immediately before transfer to the MRI scanner, coinciding with estimated peak plasma concentration (Sauer *et al.*, 2005). Mean concentration was 327 ng/ml after atomoxetine (range 147–516 ng/ml) and 0 ng/ml after placebo.

Twenty healthy age- and sex-matched controls with no history of significant neurological or psychiatric disorder participated in one session with no drug treatment, to provide normative data on performance and imaging. The study was approved by the local research ethics committee, and exempted from Clinical Trials status by the UK Medicines and Healthcare products Regulatory Authority. Participants gave written informed consent. For participants’ demographic and clinical features, see Table 1 (full medication details in Supplementary Table 1).

**Table 1:** Demographic details of participants and clinical features of patients

Features / measures	Parkinson’s disease	Control	Group difference
Number of males/females	13/6	10/10	ns
Age	69.38 (5.36)	67.40 (7.86)	ns
Years of education	12.67 (2.44)	14.47 (3.09)	ns
MMSE	28.50 (1.38)	29.30 (1.03)	ns
Years of disease	9.79 (4.91)	–	–
UPDRS (‘ON’)			
Mentation, behaviour and mood	3.16 (1.83)	–	–
Motor	25.87 (8.94)	–	–
Modified Hoehn and Yahr	2.26 (0.50)	–	–
Schwab and England Activities of Daily Living Scale	0.82 (0.15)	–	–
Levodopa equivalent daily dose	1080.16 (584.03)	–	–

Data are presented as (means, SD, and group differences). Group difference *P*-values refer to two-tailed *t*-tests or chi-squared (ns = not significant, *P* > 0.05 uncorrected). The UPDRS motor subscale (part III) was assessed on both sessions, and the average presented here. The levodopa equivalent daily dose was estimated according to Tomlinson *et al.* (2010).

### Stop-signal task

Subjects performed a response inhibition task during functional MRI, described in detail previously (Ye *et al.*, 2014, 2015). In brief, there were 360 ‘Go’ trials (75%), 80 ‘stop’ trials (17%), and 40 ‘NoGo’ trials (8%), in a randomized order. On Go trials, subjects responded to a left or right black arrow (1000 ms) with their right hand, followed by an intertrial interval with a fixation cross on blank background. On stop trials, the initial Go cue was replaced after a variable ‘stop-signal delay’ by a red arrow (<1500 ms) and auditory tone. The stimulus onset asynchrony was 2500 ms. The stop-signal delay was varied by an online algorithm (increment 50 ms) to maintain successful inhibition on 50% of trials. On NoGo trials, a red arrow and auditory tone appeared at trial onset (equivalent to a stop-signal delay of 0 ms). Given the preclinical evidence of noradrenergic regulation of action cancellation, and frequent use of stop-signal paradigms in other disorders and comparative studies, we focus our analysis on the stop trials. The stop-signal reaction time (SSRT) was calculated by the integration method (Logan and Cowan, 1984), adjusting for Go trial omissions (Ye *et al.*, 2015). We expected individual differences in performance deficits (Obeso *et al.*, 2011*a*; Ye *et al.*, 2015), correlating ‘baseline’ SSRT on placebo and the extent of improvement on atomoxetine (Pearson’s correlation, SPSS).

### MRI acquisition

Functional MRI data were acquired using T_2_*-weighted echo-planar imaging (Siemens Trio 3T, 2000 ms repetition time, 30 ms echo time, 192 × 192 mm^2^ field of view, 32 sequential descending axial slices, 3 mm slice thickness, 0.75 mm gap, 3 × 3 mm^2^ in-plane resolution). Eleven initial images were discarded to allow steady-state magnetization. Five hundred and twenty-five milliseconds after the start of the 12th image, the task began with a fixation cross of 500-ms duration, before the first arrow stimulus onset. A magnetization-prepared rapid acquisition gradient echo (MPRAGE) T_1_-weighted structural image was acquired for co-registration and normalization (2300 ms repetition time, 2.86 ms echo time, 1.25 × 1.25 × 1.25 mm resolution).

To exclude significant drug-induced changes in cerebral perfusion, we used pulsed arterial spin labelling (PASL) after the stop-signal task (PICORE-Q2T-PASL, 2500 ms repetition time, 13 ms echo time, 256 × 256 mm^2^ field of view, nine slices, 8 mm slice thickness, 2 mm gap, flip angle 90°, 700 ms inversion time 1, 1800 ms, inversion time 2 first slice, 1600 ms saturation stop time, 100 mm tag width and 180 mm gap, 90 repetitions giving 45 tag–control pairs). A single-shot EPI (M0) equilibrium magnetization scan was acquired.

A diffusion-weighted sequence was acquired with 63 directions (b = 1000 s/mm^2^, 7800 ms repetition time, 90 ms echo time, 63 sequential interleaved ascending axial slices, 192 × 192 mm^2^ field of view, 2 mm slice thickness and 2 × 2mm^2^ in-plane resolution). For patients, we acquired diffusion-weighted data once, usually on the first session. Half of patients’ diffusion data were collected on placebo, and half on atomoxetine. LEDD did not correlate with fractional anisotropy (Rae *et al.*, 2012). Diffusion data were unavailable for two control subjects.

### Functional MRI: preprocessing and statistical modelling

Functional MRI preprocessing and analysis used SPM8 (r5236; www.fil.ion.ucl.ac.uk/spm) with DCM10 (r5236) and Automatic Analysis scripts (AA4, https://github.com/rhodricusack/automaticanalysis). Functional images were realigned to the mean image and sinc interpolated in time to the middle slice. The MPRAGE was co-registered to the mean functional image, and segmented and normalized to the SPM MNI152 template. The normalization parameters were applied to functional images, before smoothing with a Gaussian kernel of full-width at half-maximum 8 mm.

Single-subject first-level general linear models were used to model task events and moderator terms for DCM, while accounting for experimental variance across the different trial types. Events were modelled with 1 s duration, convolved with the canonical haemodynamic response function. Go, Stop-correct, Stop-incorrect, and NoGo-correct trials were present for every subject. NoGo-incorrect, Go-error or Go-omission trials were modelled if the individual made incorrect responses. The first column of the design matrix included all trial types. The second, third, and fourth regressors were parametric modulators of ‘all trials’ as follows: ‘stopping’, represented by Stop-correct > Go; ‘no-going’, represented by NoGo-correct > Go; ‘stop accuracy’, represented by Stop-correct > Stop-incorrect. Serial orthogonalization was not applied when estimating the first-level general linear models. Six nuisance regressors modelled subject movement as three translations and three rotations.

To examine group effects, a second-level SPM analysis used contrasts of interests from the first level (Stop-correct > Go). Group-level statistical maps were calculated for (i) controls; (ii) Parkinson’s disease (PD)-placebo; and (iii) PD-atomoxetine, with one-sample *t*-tests. Then, group differences between the controls and PD-placebo (‘disease effect’, including also the effect of dopaminergic medication and placebo medication) were examined using two-sample *t*-tests. To examine the effect of atomoxetine, we compared within-group differences between PD-placebo and PD-atomoxetine sessions using a repeated-measures general linear model (‘atomoxetine effect’). Contrasts were corrected for multiple comparisons with cluster-based false discovery rate at *P* < 0.05 (FDRc, Chumbley and Friston, 2009) or at an exploratory threshold *P* < 0.001 uncorrected, with >50 voxels per cluster. Local maxima are listed in Supplementary Table 2.

### Dynamic causal modelling

Dynamic causal modelling is a hypothesis-driven method to quantify directional influences among brain regions (Friston *et al.*, 2003), inverting a set of biologically plausible generative brain network models to the observed data (Stephan *et al.*, 2010). For each model, the free energy estimate of the log-evidence (F) provides a measure of model accuracy adjusted for complexity (Stephan *et al.*, 2010).

To assess the response inhibition network we inverted the 20 models from Rae *et al.* (2015). These varied the interactions between the inferior frontal gyrus, preSMA, subthalamic nucleus and motor cortex. We selected these regions given their essential contributions to action stopping (Chambers *et al.*, 2006; Aron *et al.*, 2014; Rae *et al.*, 2015), and their dysfunction during action control in Parkinson’s disease (Rowe *et al.*, 2010; Ray *et al.*, 2012; Ye *et al.*, 2015). We estimated connectivity between brain regions in terms of (i) the ‘average’ connectivity as a function of external task-based perturbation on all trials. This is a weighted average across different event types (DCM.A matrix), sometimes known as baseline, fixed or the average connectivity (Stephan *et al.*, 2008; Rowe *et al.*, 2010; Rae *et al.*, 2015); (ii) the modulatory influences on connections associated with successfully stopping an action (DCM.B matrix in bilinear models) or activity in other regions (DCM.D matrix, non-linear models only); and (iii) inputs that drive network activity (DCM.C matrix, on ‘all trials’).

To model connectivity, we extracted the first eigenvariate of functional MRI time series in 5-mm spheres from each subject’s F-map, in left primary motor cortex (M1), subthalamic nucleus, preSMA, and right inferior frontal gyrus. F-maps were thresholded at *P* < 0.05 to identify the local maximum closest to the group peak that also conformed to appropriate regional anatomy. The group peak coordinates were defined from a second-level analysis including control and PD-placebo session data using a one-sample *t*-test of ‘all trials’ for primary motor cortex (*x* −38, *y* −20, *z* 60), and a one-sample *t*-test of ‘stopping’ for preSMA (*x* 8, *y* 16, *z* 56) and right inferior frontal gyrus (*x* 46, *y* 16, *z* 28). The right inferior frontal gyrus peak was used due to right hemisphere lateralization in response inhibition tasks (Aron *et al.*, 2014; Rae *et al.*, 2014), and greater right inferior frontal gyrus activation during stopping at the second-level in both patients and controls. The subthalamic nucleus region of interest used a mask from the probabilistic maps from Forstmann *et al.* (2012): 57 voxels of 2 mm^3^ (Rae *et al.*, 2015).

The 20 models comprised four ‘families’. Families differed first according to their weighted average connectivity (DCM.A) between inferior frontal gyrus and preSMA, which could be absent, unidirectional, or bidirectional (Fig. 2). This variation in model structure permits inference on how the inferior frontal gyrus and preSMA interact during stopping. Second, we varied the modulatory effect of action stopping, which represented successful stopping (parametric modulator Stop-correct > Go: Fig. 2). Dynamic causal models can be ‘linear’ (Friston *et al.*, 2003) or ‘non-linear’ (Stephan *et al.*, 2008). We compared 12 linear models, in which successful stopping directly modulated the strength of connectivity between brain regions (DCM.B). We also compared eight non-linear models, in which a frontal region (e.g. preSMA) served as the modulatory influence of connectivity between two other regions, thereby gating connectivity to the subthalamic nuclei from the other prefrontal region (DCM.D). DCM includes condition-specific inputs that drive the network dynamics. Driving inputs were applied to both the inferior frontal gyrus and preSMA (Fig. 2). Models were inverted using DCM10 (r5236) with default prior distributions for haemodynamic and coupling parameters, Gaussian error terms, and liberal prior variances on self-connections (permitting a broad range of neuronal transients up to several seconds), connection parameters (reducing the probability of excursions into unstable domains of parameters space during optimization) and hyperparameters. These match typical evoked neurophysiological responses and our task design of short discrete events support accurate estimation of connection strengths over a wide range of observation noise (Friston *et al.*, 2003).

**Figure 1:**
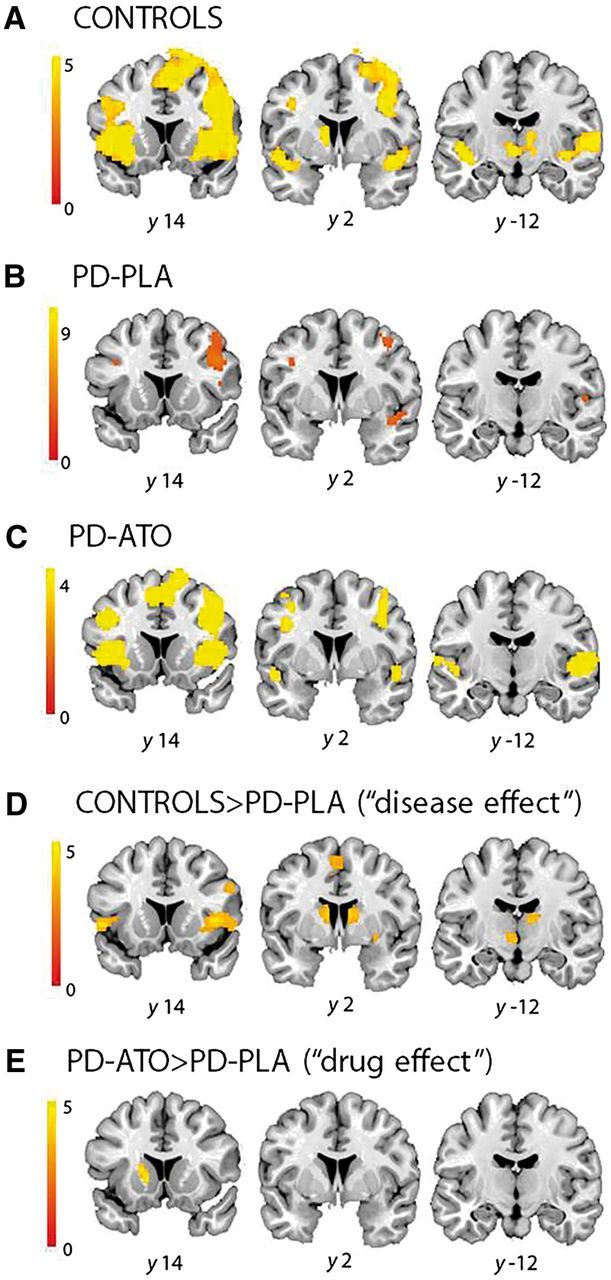
**Stopping activations (Stop-correct > Go).** (**A**) Controls; (**B**) PD-placebo; (**C**) PD-atomoxetine (ATO); (**D**) controls > PD-placebo (PLA, ‘disease effect’); and (**E**) PD-atomoxetine > PD-placebo (‘drug effect’). (**A–D**) Shown at *P* < 0.05 FDR-cluster corrected; (**E**) shown at *P* < 0.001 uncorrected with minimum 50 voxels per cluster (no significant clusters at *P* < 0.05, FDRc). Note that the ‘disease effect’ incorporates the presence of Parkinson’s disease, the use of usual dopaminergic medications in patients (but not in controls), and the session-specific use of a placebo tablet. The ‘drug effect’ refers only to the main effect of atomoxetine, which may obscure significant individual patient differences in regional brain activations.

**Figure 2:**
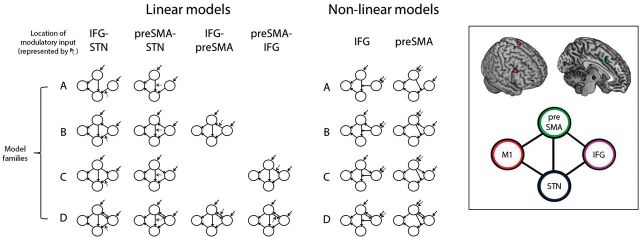
**The 20 causal models.** Each represents alternative hypotheses of frontal-subcortical interactions during response inhibition (from Rae *et al.*, 2015). We compared which of these models was most likely for controls, PD-placebo, and PD-atomoxetine (see Fig. 3). The *inset* panel shows the schematic arrangement of the four model regions [preSMA, inferior frontal gyrus (IFG), subthalamic nucleus (STN), M1] in each of the 20 models. In ‘linear’ models, successful stopping modulates the strength of connectivity between brain regions. In ‘non-linear’ models, stopping modulates activity of one region (e.g. preSMA), which gates the strength of connectivity from the other frontal region (e.g. inferior frontal gyrus) to the subthalamic nucleus. Dotted arrows indicate the location of the modulatory effect of stopping in each model. Solid arrows indicate exogenous driving inputs of all task trials. Model ‘families’ (**A–D**) share average connectivity patterns between the two frontal regions (preSMA and inferior frontal gyrus).

We compared model evidences to identify the most likely causal model of the observed functional MRI data for controls, PD-placebo, and PD-atomoxetine. We used Random Effects and Fixed Effects Bayesian Model Selection with uniform priors over model space (Bayesian Model Selection, Stephan *et al.*, 2009*a*). By convention, a difference ΔF > 5, equivalent to a Bayes factor of 150, is considered very strong evidence for the more likely model (Kass and Raftery, 1995). The Random Effects method estimates the exceedance probability (XP), which represents the probability that a given model is more likely than any other model to have generated the observed data, under the assumption that subjects may have generated data according to different generative networks (Stephan *et al.*, 2009*a*). In addition, the protected exceedance probability (Rigoux *et al.*, 2014) quantifies the likelihood that any model is more frequent than the others, above and beyond chance (Table 2). The Fixed Effects analysis provides the posterior model probability, which represents the probability that a given model generated the observed group data (ranging from 0 to 1), assuming that data are generated from a common model for a group. It is potentially vulnerable to an extreme outlier’s influence on the group inference. One patient had a DCM.D parameter value >2 standard deviations (SD) from the group mean on the placebo session. We repeated model selection without this patient. There was a negligible change in the difference in F between the first and second most likely models (4.11 to 4.16).

**Table 2:** Summary of model comparisons

Group	Comparison	XP	pXP	BOR
Controls	Linear	0.0003	N/A	
	Non-linear	**0.9997**		
	IFG stopping input	0.1574	N/A	
	PreSMA stopping input	**0.8426**		
	NAp	0.0116	0.0223	0.04
	NBp	0.0423	0.0516	
	NCp	**0.9459**	**0.9146**	
	NDp	0.0002	0.0114	

PD-placebo	Linear	0.0765	N/A	
	Non-linear	**0.9235**		
	IFG stopping input	0.0129	N/A	
	PreSMA stopping input	**0.9871**		
	NAp	0.6281	0.4175	0.56
	NBp	0.1641	0.2119	
	NCp	0.2054	0.2302	
	NDp	0.0024	0.1403	

PD-atomoxetine	Linear	0.1350	N/A	
	Non-linear	**0.8650**		
	IFG stopping input	0.1234	N/A	
	preSMA stopping input	**0.8766**		
	NAp	0.4450	0.3365	0.56
	NBp	0.1487	0.2050	
	NCp	0.4033	0.3180	
	NDp	0.0030	0.1404	

Exceedance probabilities (XP) from the three-step hierarchical model selection, comparing (i) linear versus non-linear models; (ii) non-linear inferior frontal gyrus versus preSMA models; (iii) the four non-linear preSMA models with different average connectivity ‘family’ status. The protected exceedance probabilities (pXP) show that for the patient sessions, no model is more frequent than any others, above and beyond chance, reflecting the presence of marked patient heterogeneity in response to drug (Fig. 4). BOR = Bayesian Omnibus Risk.

**Figure 3:**
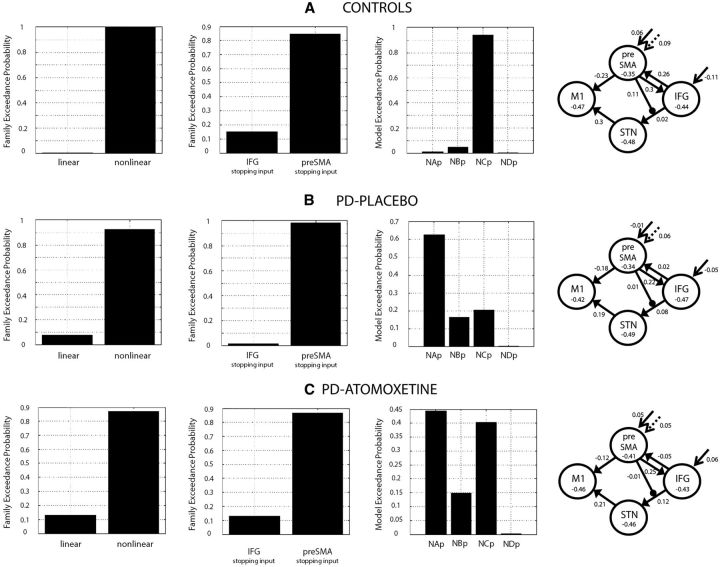
**Bayesian model selection.** Model evidences were compared across the 20 models (see Fig. 2) for (**A**) controls, (**B**) PD-placebo, and (**C**) PD-atomoxetine. In a three-step hierarchical model selection, the 12 linear models were first compared to the eight non-linear models. With evidence in favour of the non-linear models (Table 2), we next compared the non-linear inferior frontal gyrus models to the non-linear preSMA models. Finally we compared the four non-linear preSMA models according to their average connectivity ‘family’ status (see Fig. 2, and Table 2 for BOR and pXP values). Displayed to the right, for each group the model parameters are shown after Bayesian Model Averaging across the four non-linear preSMA models. Labels on the *x*-axis indicate the identity of the models as for Fig. 2: N = non-linear; A to D = model families.

In a three-step hierarchical model selection procedure (Fig. 3), we first compared the 12 linear models to the eight non-linear models. With evidence in favour of the non-linear models (Table 2), we next compared the non-linear inferior frontal gyrus models to the preSMA models. Finally we compared the four non-linear preSMA models according to their average connectivity ‘family’ status (Fig. 2).

When the exceedance probability does not exceed 0.9, it is recommended to average all models with a probability of >0.05 (Penny *et al.*, 2010). This being the case for the PD-placebo and PD-atomoxetine sessions, we used Bayesian Model Averaging over the four non-linear preSMA models, and examined the averaged model parameters in terms of individual patient differences in disease severity and drug level.

### Individual differences in frontal- subcortical connectivity

Individual differences in functional connectivity are influenced by structural connectivity (Rae *et al.*, 2015) and neurotransmitter levels (Stephan *et al.*, 2009*b*). We therefore tested whether disease severity and blood plasma levels of atomoxetine influence functional connectivity.

For each patient, we examined the averaged model connectivity parameters from the four non-linear preSMA models. The difference in parameters indicates the change in frontal-subcortical connectivity due to the drug (counterbalancing session order effects). Change in connectivity values (ΔATO-PLA) were used as dependent variables in multiple regression models (SPSS), for (i) ‘Δ inferior frontal gyrus to subthalamic nucleus connectivity’; and (ii) ‘Δ preSMA non-linear modulation’. We selected these two variables of interest as they represent the cortical inputs to the subthalamic nucleus. The independent variables in the regression models were Unified Parkinson’s Disease Rating Scale (UPDRS)-III (as a measure of disease severity) and blood plasma drug level (ng/ml).

### Pulsed arterial spin labelling: preprocessing and statistical modelling

Pulsed arterial spin labelling time series were converted to cerebral blood flow maps using ASLtbx (https://cfn.upenn.edu/∼zewang/ASLtbx.php). Following rigid-body alignment, images were smoothed with a Gaussian kernel of full-width at half-maximum 8 mm. Cerebral blood flow quantification used simple subtraction and the mean M0 signal from CSF in ventricle regions of interest for quantification. Cerebral blood flow was compared between the PD-placebo and PD-atomoxetine sessions using a whole-brain voxelwise analysis in SPM12b with a repeated measures *t*-test, corrected for multiple comparisons with family-wise error (FWE) at *P* < 0.05 and at the exploratory threshold *P* < 0.001 (uncorrected).

### Diffusion MRI

We used whole-brain tract-based spatial statistics (TBSS, Smith *et al.*, 2006) to examine correlations between fractional anisotropy and the difference in SSRT between placebo and atomoxetine sessions (ΔSSRT), complementing the analysis of functional connectivity. Using FSL 4.1.8 (www.fmrib.ox.ac.uk/fsl), diffusion images were corrected for head movements and eddy currents then smoothed with a 2.5 mm Gaussian kernel, before fitting diffusion tensors. In tract-based spatial statistics, subjects’ fractional anisotropy maps were registered to the most representative subject, and normalized to MNI152 space. A mean fractional anisotropy skeleton was created and thresholded at fractional anisotropy > 0.25. Using FSL ‘randomise’, 10 000 permutations with Threshold-Free Cluster Enhancement (TFCE, Smith and Nichols, 2009) tested for correlations between fractional anisotropy and ΔSSRT across the mean fractional anisotropy skeleton. In the design matrix, additional columns containing control SSRT, patient age, control age, UPDRS, and LEDD were entered as covariates. We also tested for correlations between fractional anisotropy and LEDD. In three separate design matrices, we tested for correlations between fractional anisotropy and the three DCM parameters representing the combined effect of the non-linear frontal input to the subthalamic nucleus (DCM.A preSMA to inferior frontal gyrus, DCM.A inferior frontal gyrus to subthalamic nucleus, and DCM.D modulation), in the most likely model for (i) controls; (ii) PD-placebo; and (iii) PD-atomoxetine. All variables were demeaned. Voxels with significant correlation between fractional anisotropy and ΔSSRT were back-projected to native space using FSL ‘deproject’.

## Results

### Behavioural performance

**Figure 4:**
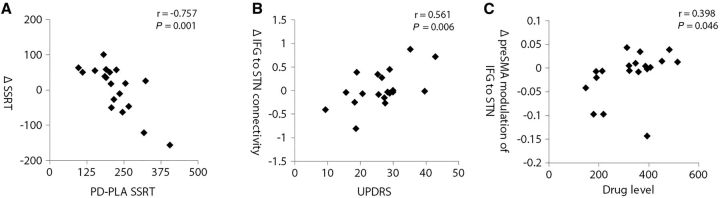
**Correlations between behaviour, UPDRS, drug level, and connectivity on atomoxetine.** (**A**) Correlation between ‘baseline’ SSRT on placebo, and change in SSRT on atomoxetine (Pearson correlation, r = −0.757, *P* < 0.001). (**B**) Correlation between UPDRS (part III, motor subscale averaged across sessions), and the change caused by atomoxetine (Δ) in connectivity from inferior frontal gyrus to subthalamic nucleus (DCM.A matrix), after Bayesian Model Averaging (Pearson correlation, r = 0.561, *P* = 0.006). (**C**) Correlation between plasma drug level (ng/ml), and change in non-linear modulation of inferior frontal gyrus to subthalamic nucleus connectivity by the preSMA (DCM.D matrix), after Bayesian Model Averaging (Pearson correlation, r = 0.398, *P* = 0.046).

Patients were impaired on the stop-signal task, with longer SSRTs on placebo (224 ms) than healthy controls (171 ms, t = −2.758, *P* = 0.009). Atomoxetine did not significantly alter the group average SSRT (228 ms, t = −0.305, *P* = 0.764) in line with previous studies (Ye *et al.*, 2015). However, the non-significant group effect obscures significant individual differences related to disease severity, neural structure and function. The baseline (placebo) SSRT correlated (r = −0.757, *P* < 0.001) with change in SSRT on atomoxetine (i.e. SSRT-placebo − SSRT-atomoxetine), indicating that ‘baseline’ SSRT was predictive of the extent to which patients improved on the drug (Fig. 4A).

### Univariate functional MRI of successful response inhibition: Stop-correct > Go

Figure 1A confirms that the healthy controls have activation of the right inferior frontal gyrus, bilateral preSMA, and bilateral caudate nucleus. Patients with Parkinson’s disease also show activation of these regions (Fig. 1B), albeit to a lesser degree: the comparison of controls versus PD-placebo (‘disease effect’) confirms significant underactivation of the right inferior frontal gyrus, preSMA, putamen and posterior caudate nucleus (Fig. 1D). The group difference in the subthalamic nucleus was significant using a small volume correction from the subthalamic nucleus map (*P* < 0.05 FWE, peak local maxima *x* −8, *y* −16, *z* −8).

On atomoxetine, patients showed activation of the right inferior frontal gyrus and bilateral preSMA (Fig. 1C). Although preSMA activation was not present in PD-placebo (Fig. 1B), a within-group contrast comparing PD-placebo versus PD-atomoxetine (‘atomoxetine effect’) did not show a significant difference (*P* > 0.05, FDRc). The exploratory threshold (*P* < 0.001) revealed activation in the left striatum on atomoxetine (Fig. 1E). Supplementary Table 1 reports local maxima for each contrast. Although atomoxetine had only modest effects on activation within regions, this does not imply a lack of effect on coupling between regions, the principal focus of this study to which we turn next.

### Structure and connectivity of the stopping network

Twenty models examined interactions among the inferior frontal gyrus, preSMA, subthalamic nucleus, and primary motor cortex during response inhibition (Fig. 2). Bayesian model selection (Stephan *et al.*, 2009*a*) compared these response inhibition network models separately for (i) controls; (ii) PD-placebo; and (iii) PD-atomoxetine (Fig. 3 and Supplementary Fig. 1). In a hierarchical model selection (Fig. 3), for all groups, evidence for the non-linear models outweighed evidence for the linear models, and for the non-linear preSMA models over the inferior frontal gyrus models (Table 2). The addition of a driving input of ‘Stop-correct > Go’ to inferior frontal gyrus or preSMA in the linear models (as exists in the non-linear models) was associated with lower model evidences (not shown).

In controls, amongst the four non-linear preSMA models, the most likely was model NCp (exceedance *P* > 0.9 and protected exceedance *P* > 0.9). In this model, successful stopping modulates activity of the preSMA, which gates the strength of inputs from the inferior frontal gyrus to the subthalamic nucleus. In addition to this non-linear modulation, there is a direct cortico-cortical influence from the preSMA to the inferior frontal gyrus (see Family C, Fig. 2).

In PD-placebo and PD-atomoxetine, no single model had an exceedance probability > 0.9, which may be due to heterogeneity in the patient group. Comparing all 20 models (Supplementary Fig. 1), the numerically most likely models in terms of exceedance probability were model NAp for PD-placebo, and model NCp (*c.f.* controls) for PD-atomoxetine. This is weak evidence, but consistent with the interpretation that in some patients with Parkinson’s disease on placebo (plus their usual dopaminergic medication), the difference to healthy controls lies in the absence of the cortical interaction from the preSMA to the inferior frontal gyrus (Family A, Fig. 2), while atomoxetine restored the most likely model to that observed in healthy controls, including the cortical interaction from the preSMA to the inferior frontal gyrus (Family C, Fig. 2).

At the group level there was no overall most likely model for either PD-placebo or PD-atomoxetine sessions (exceedance *P* < 0.9 and protected exceedance *P* < 0.9). This is consistent with the presence of subgroups of patients in a separate cohort as observed by Ye *et al.* (2015, 2016): on the placebo session, some patients exhibit a response inhibition network similar to that of controls, while others are missing the key cortico-cortical interaction within the network, and are so placed to benefit from the effect of atomoxetine. This highlights the impact of individual patient differences on neural and behavioural responses to atomoxetine. Accordingly, when there is no overall most likely model, we used Bayesian Model Averaging over the four non-linear preSMA models (Penny *et al.*, 2010; Stephan *et al.*, 2010), and extracted the model parameters to test for relationship with disease severity and plasma drug level.

To illuminate why the NAp and NCp models are the most likely in terms of their features, we examined the parameter conditional probabilities (DCM.Pp), after Bayesian Parameter Averaging across the subjects in each group. These represent the probability that a parameter has a non-zero value (0 < *P* < 1). Two parameters showed a clear difference across groups: (i) the preSMA to inferior frontal gyrus connection had a conditional probability of 0.99 in controls, and 0.94 in PD-atomoxetine, but was not present in PD-placebo (this is the connection restored by atomoxetine in a subset of patients according to Bayesian Model Selection); and (ii) the conditional probability of the stopping input to preSMA was 0.97 in controls, and 0.99 in PD-atomoxetine, but 0.57 in PD-placebo.

### Individual differences in frontal- subcortical connectivity

We used multiple regressions to test how disease severity and drug level influence frontal-subcortical connectivity.

We examined the change in connectivity values (ΔATO-PLA) between PD-placebo and PD-atomoxetine in the connectivity parameters from Bayesian Model Averaging of the four non-linear preSMA models. Disease severity (UPDRS) and drug level were entered as independent variables in multiple regression models with (i) ‘Δ inferior frontal gyrus to subthalamic nucleus connectivity’ (DCM.A); and (ii) ‘Δ preSMA non-linear modulation’ (DCM.D) as dependent variables. In the first model the UPDRS was related to the change in connectivity (β = 0.535, t = 2.419, *P* = 0.028; Pearson r = 0.561, *P* = 0.006), indicating that the greater the disease severity, the greater the change in frontal-subcortical connectivity with atomoxetine (Fig. 4B). In the second model the drug level correlated with change in preSMA modulation (β = 0.443, t = 1.817, *P* = 0.088; Pearson r = 0.398, *P* = 0.046), suggesting that the higher blood plasma levels of atomoxetine, the greater the change in modulation of frontal-subcortical connectivity by the preSMA (Fig. 4C).

### Perfusion and haemodynamics

A repeated-measures ANOVA comparing pulsed arterial spin labelling measures of cerebral blood flow between the PD-placebo and PD-atomoxetine sessions showed no effects of atomoxetine on local perfusion at corrected or uncorrected thresholds. This suggests that changes in activation and connectivity were more likely to be due to an effect of atomoxetine on neuronal interactions, rather than on perfusion. The haemodynamic parameters estimated by DCM model inversion provide an alternative means to assess the potential impact of drug on neurovascular responses. In a repeated-measures ANOVA, the estimated transit, decay and epsilon parameters of the haemodynamic balloon model did not differ between placebo and atomoxetine sessions (F < 1).

### Diffusion MRI

**Figure 5:**
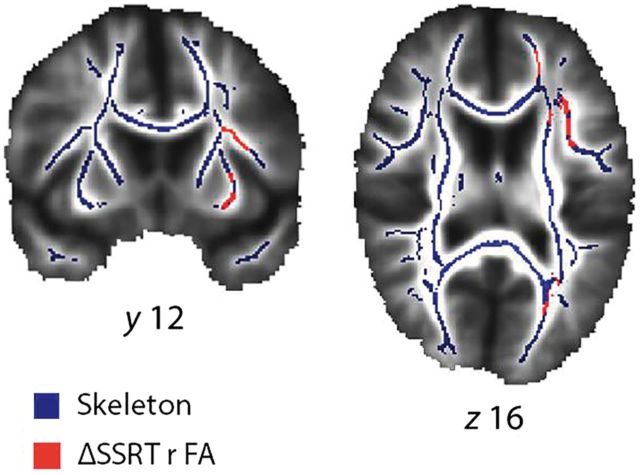
**White matter structure correlates with response inhibition.** The change in stopping efficiency (ΔSSRT) between PD-placebo and PD-atomoxetine sessions correlates with fractional anisotropy in white matter underneath the inferior frontal gyrus and in the external capsule (*P* < 0.05 TFCE corrected).

Tract-based spatial statistics confirmed a significant correlation between change in SSRT on atomoxetine (versus placebo) and fractional anisotropy in white matter underlying the inferior frontal gyrus and external capsule (*P* < 0.05 TFCE corrected, Fig. 5). At a lower threshold (*P* < 0.055 TFCE corrected), there was a trend correlation between fractional anisotropy and change in SSRT in the internal capsule, which carries interactions between frontal cortex and subcortical nuclei, previously shown to be abnormal in Parkinson’s disease (Rae *et al.*, 2012; Ye *et al.*, 2015). These results confirm that white matter structure in frontal-subcortical pathways influences the degree of behavioural change in response to atomoxetine (Ye *et al.*, 2015).

In separate permutation analyses, we tested for correlations between fractional anisotropy and the three DCM parameters representing the combined effect of the non-linear frontal input to the subthalamic nucleus (DCM.A preSMA to inferior frontal gyrus, DCM.A inferior frontal gyrus to subthalamic nucleus, and DCM.D modulation). None of these showed significant (*P* < 0.05 TFCE) correlations between fractional anisotropy and DCM parameters.

## Discussion

In Parkinson’s disease, the ability to stop actions is impaired, together with abnormal connectivity in the response inhibition network. We found that atomoxetine can enhance the interaction between the preSMA and inferior frontal gyrus, two regions that act together to influence the subthalamic nucleus for successful response inhibition. Moreover, atomoxetine increased the efficiency of stopping (ΔSSRT) in proportion to structural connectivity of white matter likely carrying interactions between frontal cortex and the subthalamic nuclei. This network approach builds on prior reports of atomoxetine’s influence on regional activation (Ye *et al.*, 2015), confirming the importance of disease severity and drug levels such that atomoxetine only changes connectivity and improves performance in a subset of patients (Kehagia *et al.*, 2014; Ye *et al.*, 2015, 2016). When atomoxetine does improve response inhibition, it does so with enhanced functional network connectivity: the effect is more likely in people with poorer baseline inhibitory control who have an optimum drug level, moderately severe disease and ‘relatively’ intact white matter.

### The response inhibition network in Parkinson’s disease

Interactions between the preSMA, inferior frontal gyrus, and their projections to the subthalamic nucleus are critical for response inhibition (Aron *et al.*, 2003; Chambers *et al.*, 2006; Frank *et al.*, 2007; Ballanger *et al.*, 2009; Duann *et al.*, 2009; Forstmann *et al.*, 2012; Rae *et al.*, 2015). Multi-stage pathways between these regions underpin the control of action, through direct, indirect, and hyperdirect routes (Nambu *et al.*, 2002; Redgrave *et al.*, 2010; Wiecki and Frank, 2013).

Patients differed from controls in the influence of preSMA on the inferior frontal gyrus: in some patients, this input was missing, but restored by atomoxetine. The relevance of this connection to model selection is suggested by the conditional probabilities in both Random Effects and Fixed Effects comparisons (the DCM.Pp values). These also accord with the univariate analyses in Fig. 1, such as reduced activation of preSMA in patients on placebo. However, model comparison should properly be made on the evidence for the whole model, because of potential covariances among parameters (Rowe *et al.*, 2010).

In older adults, as in younger adults (Rae *et al.*, 2015), these areas interacted in the influence of their projections to the subthalamic nucleus, to successfully stop an action. The non-linear interaction between subcortical projections of the preSMA and inferior frontal gyrus on the subthalamic nucleus differed between the elderly controls in this study and that previously observed in healthy young adults (Rae *et al.*, 2015). However, the leading models here and in Rae *et al.* (2015) shared the critical feature of the non-linear interaction of the cortical projections to subthalamic nucleus. These winning models are distinct from the models embodying precedence of preSMA or inferior frontal gyrus in the stopping process. We do not infer an effect of ageing on the stopping network from these data: to resolve potential cohort effects other than age would require epidemiological selection of old and young (Shafto *et al.*, 2014). Moreover, age would not explain the difference between groups in the current study, as patients were compared to age-matched controls.

The subthalamic nucleus acts as a ‘brake’ on thalamocortical outputs while the best course of action is determined (Frank *et al.*, 2007; Wiecki and Frank, 2013). Deep brain stimulation at the subthalamic nucleus improves bradykinesia in Parkinson’s disease via stabilization of dysfunctional cortico-subcortical oscillations. However, there is mixed evidence for the impact of DBS on impulsivity (Ballanger *et al.*, 2009; Mirabella *et al.*, 2012), which may not restore a window of opportunity for this brake function in situations of response conflict (Frank *et al.*, 2007). Together, these data suggest the restorative effect of atomoxetine occurs upstream of the basal ganglia, in the frontal cortex (*c.f.*Borchert *et al.*, 2016).

Our results suggest that atomoxetine exerts its effect at the level of the inferior frontal gyrus, increasing sensitivity to afferent inputs from the preSMA and activation on successful stop trials. This interpretation is supported by the anatomical distribution of a1- and a2-noradrenergic receptors, and noradrenaline transporters, which are scarce in the basal ganglia, but dense in frontal cortex (Logan *et al.*, 2007; Amunts *et al.*, 2010).

Other neurotransmitter systems also modulate the effective connectivity in motor and inhibition networks. For example, Herz *et al.* (2015) used DCM to show that dopaminergic treatment modulates the connectivity from putamen to motor cortex, including pathways via subthalamic nucleus, during response inhibition trials. In addition, the effect of serotonergic enhancement by citalopram depends in part on the integrity of the anterior limb of the internal capsule that contains the direct and indirect projections from the frontal cortex to the subthalamic nucleus (Ye *et al.*, 2014). The effects of dopamine and serotonin were increased in those with levodopa-induced dyskinesia or more advanced disease, respectively. Combinations of noradrenergic, dopaminergic and serotonergic approaches may be clinically advantageous (Huot *et al.*, 2015) at the expense of understanding the individual contribution of each monoamine and links to preclinical models (Eagle *et al.*, 2008; Bari *et al.*, 2011).

### The impact of individual differences

Atomoxetine did not improve patients’ response inhibition performance at the group level, in contrast to Kehagia *et al.* (2014) but in keeping with Ye *et al.* (2015). In addition, while there was a clear model comparison result in the control group, there was evidence for subgroups of patients: on the placebo session, some patients exhibit a response inhibition network similar to that of controls, while others are missing the key cortico-cortical interaction within the network, and so are placed to benefit from the effect of atomoxetine. This is highlighted by the Random Effects model selection analysis applying protected exceedance probabilities (Rigoux *et al.*, 2014) in which, at the group level, one considers how likely it is that any model is more frequent than the others, above and beyond chance, in contexts in which there are potentially differences in model parameters in a common network or differences in the underlying network architecture between subjects. For these reasons, we examined associations with individual patient characteristics and changes in connectivity. Individual differences in Parkinson’s disease severity, drug levels, and white matter structure of frontal-subcortical tracts influence cognitive and behavioural dysfunction including response inhibition (Chamberlain *et al.*, 2007; Ye *et al.*, 2015). In our cohort, we confirmed that disease severity and drug plasma levels relate to frontal-subcortical connectivity in response inhibition; and that structural frontal-subcortical connectivity correlated with the effect of atomoxetine on performance (ΔSSRT).

Diffusion tensor imaging reveals that the white matter underlying the inferior frontal gyrus is abnormal in Parkinson’s disease, including the tracts connecting frontal cortex to the basal ganglia (Rae *et al.*, 2012; Agosta *et al.*, 2013). This may relate to loss of axonal projections through secondary degeneration, changes in myelin, or reduced axonal calibre (Wheeler-Kingshott and Cercignani, 2009; Rae *et al.*, 2012). It means that enhancement of cortical activity during response inhibition may be unable to exert a behavioural effect because of functional disconnection from the basal ganglia. The cortical influences on the subthalamic nucleus are not absent, but the white matter change is sufficient to undermine drug efficacy: the greater the fractional anisotropy in the white matter underlying the inferior frontal gyrus, the greater the change in SSRT between placebo and atomoxetine sessions.

We propose that patients with ‘relatively’ preserved white matter are better able to transform cortical responses into behaviour. Although both noradrenergic deficits and anatomical change are progressive, individual differences in the rates at which these systems change influence behavioural change and treatment response. There is a precedent for this in dopaminergic systems, in which the dopaminergic effects on function depends on cortico-subcortical anatomical connectivity (van der Schaaf *et al.*, 2013; van Schouwenburg *et al.*, 2013).

Individual differences in drug levels may result from genetic variation in atomoxetine catabolism and variation in the impact of Parkinson’s disease pathology on visceral and digestive function (Goedert *et al.*, 2013). It is important to consider such individual differences when evaluating atomoxetine as a therapeutic tool, and look beyond the lack of a whole-group change of behaviour. This also has implications for the stratification of heterogeneous patients in clinical trials.

### Mechanisms of action

We studied patients on their normal regimen of dopaminergic medication (i.e. in their ‘ON’ state). We did not examine the ‘OFF’ state as we anticipate that future noradrenergic therapies would be used in the context of dopaminergic therapy not instead of it. Although we emphasize the role of noradrenaline in response inhibition (Eagle and Baunez, 2010; Bari and Robbins, 2013), dopamine also influences impulsivity and the control of action (Hughes *et al.*, 2010, 2013; Cummins *et al.*, 2012; Nandam *et al.*, 2013; Cubillo *et al.*, 2014; Napier *et al.*, 2014). Despite highly selective binding to noradrenaline transporters, atomoxetine might also increase extracellular cortical dopamine (Bymaster *et al.*, 2002), by blocking reuptake of dopamine via the noradrenaline transporter (Yamamoto and Novotney, 1998). However, in contrast to the dual-action of methylphenidate, the effect of atomoxetine on noradrenaline is much greater than on dopamine (Bymaster *et al.*, 2002). Moreover, levodopa has little influence on SSRT in Parkinson’s disease (Obeso *et al.*, 2011*b*) or preclinical models.

Levodopa and dopamine agonists have been associated with impulse control disorders, such as pathological gambling, hypersexuality and binge eating (Voon *et al.*, 2009; Napier *et al.*, 2014). Currently, the main strategy to treat impulse control disorders is to reduce dopaminergic medication. However, impulsivity and poor response inhibition are common even in the absence of impulse control disorders (Nombela *et al.*, 2014*a*) and can occur before dopaminergic therapy. We speculate that the effects of atomoxetine are not mediated primarily by a dopaminergic mechanism, but that atomoxetine might facilitate other approaches to treating impulsivity, enhancing response inhibition, and more conservative approaches to risk-taking (Kehagia *et al.*, 2014).

Indirect effects of atomoxetine must also be considered, including modulation of the neurovascular coupling (Iannetti and Wise, 2007). Therefore we used perfusion arterial spin labelling, but did not find a significant effect on perfusion. It is important to note that DCM optimizes the neurovascular response function as part of model inversion, and accommodates session specific variations in the haemodynamic response. In our implementation of DCM, we used default priors for haemodynamic and coupling parameters in keeping with previous clinical applications in Parkinson’s disease (Rowe *et al.*, 2010; Kahan *et al.*, 2014; Michely *et al.*, 2015). There may be grounds to adjust these priors if, for example, pulsed arterial spin labelling indicates an altered vascular response, or when studying extreme outlier neurophysiological states (Gilbert *et al.*, 2016). However, the posterior parameters of the ‘balloon model’ of haemodynamic responses did not differ between drug sessions, consistent with a lack of effect of noradrenaline infusion on human cerebral blood flow and reactivity (Moppett *et al.*, 2008).

The model inversion and model selection procedures, in which model selection is weighted by the subjects’ model precision, provide replicable inferences of causal (directional) interactions between brain regions (Rowe, 2010; Friston *et al.*, 2013). This underlies the widespread use of DCM to study changes in functional network dynamics from disease (Rowe *et al.*, 2010; Kahan *et al.*, 2014; Michely *et al.*, 2015), remission (Goulden *et al.*, 2012), and treatment (Wang *et al.*, 2011; Herz *et al.*, 2015). Although DCM has been validated using functional MRI combined with invasive recordings in animals (David *et al.*, 2008), it is also useful to seek corroboration from other methods. For example, DCM connectivity parameters correlate with neurophysiological measures using transcranial magnetic stimulation (Boudrias *et al.*, 2012), and correlate with brain structure and function (Song *et al.*, 2013; Rae *et al.*, 2015). Here, the effects of both white matter structure and functional connectivity on behaviour support our DCM inferences.

Despite the advantages of DCM, it does not distinguish between mono- and polysynaptic pathways. Therefore, one cannot determine whether the interaction between preSMA and inferior frontal gyrus is via direct cortico-cortical pathways or through a subcortical pathway (Coxon *et al.*, 2012; Forstmann *et al.*, 2012; Rae *et al.*, 2015). The thalamus, striatum, and globus pallidus are also relevant to response inhibition (Nambu *et al.*, 2002; Redgrave *et al.*, 2010; Wiecki and Frank, 2013) and our models implicitly incorporate these subcortical stage posts. However, we did not delineate these stage posts, focusing instead on four brain regions that were necessary and sufficient to test our hypotheses (Chamberlain *et al.*, 2007; Cubillo *et al.*, 2014; Ye *et al.*, 2015). This method also guards against a computational explosion of unnecessary models, and balances model complexity and generalizability (Stephan *et al.*, 2010). Future studies however could explore further the modulation of activity within the direct, indirect, and hyperdirect pathways.

This study highlights the potential utility for atomoxetine as an adjunctive therapy in Parkinson’s disease, if stratified in light of the patient differences that influence its behavioural benefit. However, we studied an acute single dose of atomoxetine, in order to explore its effects on underlying neural mechanisms of a task that provides a direct link to preclinical studies of the neural basis of impulsivity. Clinical doses of atomoxetine may be higher, and used chronically (Clemow, 2014). Indeed, chronic atomoxetine up to 100 mg is well-tolerated in Parkinson’s disease (Marsh *et al.*, 2009). Future patient studies in larger cohorts may also wish to consider the impact of genetic variations on response inhibition ability (Cummins *et al.*, 2012), or other imaging markers of cognition in Parkinson’s disease (Nombela *et al.*, 2014*b*). For example, polymorphism of the noradrenaline transporter affects inferior frontal gyrus activation and behaviour in the stop-signal task (Whelan *et al.*, 2012).

## Conclusions

The analysis of brain network interactions provides mechanistic insights into noradrenergic influences on behaviour in Parkinson’s disease. We provide a replication of the potential for atomoxetine to improve response inhibition in some, but not all cases, according to variations in baseline performance, disease severity, drug level and white matter structure. In addition, we provide evidence that atomoxetine modulates cortico-cortical functional connectivity in Parkinson’s disease, subject to individual patient differences. These factors validate preclinical models of disease, executive function and impulsivity. They also highlight the need for stratification in clinical trials of noradrenergic therapy, as an adjunct to dopaminergic therapy, so as to direct noradrenergic treatment to the subset of patients who are most likely to benefit.

## Supplementary Material

Supplementary Data

## Abbreviations

DCM =: Dynamic Causal Modelling
preSMA =: pre-supplementary motor area
SSRT =: stop-signal reaction time
UPDRS =: Unified Parkinson’s Disease Rating Scale
