# Supplementary material for: Atomoxetine restores the response inhibition network in Parkinson’s disease
Source: Brain. 2016 Jun 24;139(8):2235–48. doi: 10.1093/brain/aww138 (PMC4958901; doi:10.1093/brain/aww138)

Atomoxetine restores the response inhibition network in Parkinson's disease

Supplementary Information

Charlotte L Rae,[1,2] Cristina Nombela,[1] Patricia Vázquez Rodríguez,[1] Zheng Ye,[1] Laura E Hughes,[1,2] P Simon Jones,[1] Timothy Ham,[1] Timothy Rittman,[1] Ian Coyle-Gilchrist,[1] Ralf Regenthal,[3] Barbara J Sahakian,[4,5] Roger A Barker,[1] Trevor Robbins,[4,6] James B Rowe,[1,2,4]

Author affiliations:

1 Department of Clinical Neurosciences, University of Cambridge, Cambridge, CB2 0SZ, UK

2 Medical Research Council Cognition and Brain Sciences Unit, Cambridge, CB2 7EF, UK

3 Division of Clinical Pharmacology, Rudolf-Boehm-Institute of Pharmacology and Toxicology, University of Leipzig, Leipzig, 04107, Germany

4 Behavioural and Clinical Neuroscience Institute, Cambridge, CB2 3EB, UK

5 Department of Psychiatry, University of Cambridge, CB2 0SZ, Cambridge, UK

6 Department of Experimental Psychology, University of Cambridge, CB2 3EB, Cambridge, UK

Correspondence to:

Professor James B Rowe

Department of Clinical Neurosciences,

University of Cambridge,

Cambridge,

CB2 0SZ,

UK james.rowe@mrc-cbu.cam.ac.uk

Supplementary Table 1. Patient disease and medication details. *LEDD calculated according to Tomlinson et al. (2010, *Mov Disord*)

**Patient**	**Disease duration**	**UPDRS III motor subscale** **(average of PD-PLA & PD-ATO sessions)**	**LEDD***	**Medications (daily dose in mg)**
1	5	9	510	Co-careldopa 300<br>Pramipexole 2.1
2	16	28	2260	Madopar controlled release 200<br>Madopar 1000<br>Ropinirole 24<br>Amantadine 300
3	1	30	450	Co-beneldopa 450
4	12	29	880	Co-careldopa 400<br>Ropinerole 24
5	4	30	800	Madopar 800
6	9	21	1704	Stalevo 800<br>Co-careldopa 400<br>Rotigotine 8
7	9	28	865	Sinemet Plus 550<br>Pramipexole 3.15
8	16	35	2450	Sinemet controlled release 200<br>Co-careldopa 800<br>Apomorphine 150
9	9	29	1912	Stalevo 750<br>Sinemet controlled release 400

				Madopar 50<br>Pramipexole 2.64
10	18	18	1098	Stalevo 600<br>Ropinirole 15
11	11	27	798	Stalevo 600
12	9	25	965	Co-beneldopa 150<br>Madopar 300<br>Pramipexole 3.15<br>Amantadine 200
13	6	27	810	Co-beneldopa 300<br>Madopar controlled release 200<br>Ropinirole 8<br>Amantadine 200
14	12	19	982	Stalevo 400<br>Sinemet controlled release 600
15	18	19	1060	Co-beneldopa 700<br>Co-beneldopa controlled release 200<br>Pramipexole 2.1
16	8	40	610	Co-careldopa 400<br>Pramipexole 2.1
17	5	26	760	Madopar 600<br>Ropinirole 8
18	5	16	450	Co-beneldopa 300

				Pramipexole 1.5
19	13	43	1159	Stalevo 450<br>Ropinirole 18<br>Amantadine 200

Supplementary Table 2. Local maxima of significant activations for the contrast Stop-correct>Go (parametric modulator), localized according to the Anatomy toolbox in SPM8. (L=left hemisphere; R=right hemisphere; x, y z = coordinates of maximum activated voxel in standard MNI152 space; *t* = *t* stat at this voxel.) Peaks for A-D are listed at $p<0.05$ FDR-cluster corrected (FDRc), peaks for E are listed at $p<0.001$ uncorrected with minimum 50 voxels per cluster (no significant clusters at $p<0.05$ FDRc).

Region	Hemisphere	MNI coordinates			*t*
		x	y	z	
(A) Controls, FDRc p<0.05					
Insula	L	-32	18	6	11.52
	R	34	24	-4	10.70
	R	44	22	0	10.43
Inferior frontal gyrus (p. opercularis)	R	38	6	30	11.17
	R	52	18	28	9.90
	R	44	14	28	9.71
Superior temporal gyrus	R	62	-42	14	10.59
	L	-64	-36	20	9.54
	R	62	-30	10	9.36
	R	60	-28	8	9.30
	L	-64	-40	14	9.10
	L	-52	-38	14	9.07
	L	-44	-18	2	8.55
	L	-58	-30	12	8.50
	L	-38	-28	6	7.79
	L	-46	-12	-6	7.74
Angular gyrus	R	28	-60	42	10.58
Inferior temporal gyrus	R	42	-68	-8	9.74
Middle temporal gyrus	L	-44	-24	0	8.94
Inferior occipital gyrus	R	34	-74	-10	8.76
	L	-36	-66	-10	7.72

	L	-42	-78	-6	7.40
	L	-44	-70	-8	7.38
Fusiform gyrus	L	-34	-70	-10	8.19
	L	-38	-54	-14	5.68
Middle occipital gyrus	L	-36	-86	8	8.14
	L	-32	-88	-4	8.06
	L	-22	-94	0	7.54
	L	-18	-104	4	4.52
Heschl's gyrus	L	-38	-30	10	7.82
Inferior frontal gyrus (p. triangularis)	L	-50	16	-2	6.73
Thalamus	L	-10	2	6	6.47
Middle frontal gyrus	L	-38	46	4	5.54
Superior orbital gyrus	L	-26	52	-4	4.83
Middle orbital gyrus	L	-40	56	-6	4.52
	L	-30	50	-8	4.45
Cerebellum	L	-14	-76	-34	4.66
	L	-24	-76	-42	4.15
(B) PD-PLACEBO, FDRc p<0.05					
Inferior occipital gyrus	L	-36	-70	-8	10.99
	L	-36	-78	-6	9.22
	R	38	-72	-8	8.91
	R	34	-82	-6	8.06
Superior temporal gyrus	L	-58	-44	12	10.53
	R	64	-34	8	7.57
	R	52	-36	14	7.29
	R	68	-24	12	6.09
	R	50	-22	-4	5.70
	L	-66	-18	2	5.51
	R	50	0	-10	4.15
	R	52	-2	-8	4.08

Inferior temporal gyrus	L	-42	-62	-8	7.40
	R	46	-56	-12	3.80
Middle temporal gyrus	L	-50	-20	-2	7.33
Inferior parietal lobule	R	32	-52	46	7.19
	R	52	-54	50	6.97
	L	-58	-56	42	5.49
	L	-60	-50	44	4.31
	L	-38	-38	20	3.96
Angular gyrus	R	28	-56	44	6.66
Supramarginal gyrus	R	48	-42	44	6.05
	R	56	-46	34	4.95
	R	60	-44	24	4.92
	L	-56	-46	30	4.21
Middle frontal gyrus	R	42	10	46	6.01
	R	36	8	38	5.19
Middle occipital gyrus	L	-24	-92	2	5.94
	R	30	-94	4	5.68
	L	-20	-96	4	5.44
Inferior frontal gyrus (p. opercularis)	R	46	18	26	5.64
	R	46	18	6	4.19
	R	44	20	8	4.10
Insula	R	32	22	6	5.18
	L	-28	22	-2	4.94
	L	-30	24	2	4.88
Inferior frontal gyrus (p. orbitalis)	R	30	24	-6	5.16
Precentral gyrus	L	-40	6	30	4.80
Cuneus	R	20	-98	8	4.78
Heschl’s gyrus	R	54	-14	8	4.26
Fusiform gyrus	L	-36	-50	-14	4.19
Inferior frontal gyrus (p. triangularis)	R	40	26	24	4.06

	R	58	24	18	3.93
	L	-34	18	10	3.63
(C) PD-ATOMOXETINE, FDRc p<0.05					
Inferior frontal gyrus (p. opercularis)	R	38	10	28	10.40
Insula	R	38	20	-4	10.18
	R	32	22	-2	9.78
	L	-28	22	-4	8.92
	L	-32	18	8	5.77
Superior temporal gyrus	R	60	-42	16	9.09
	R	64	-26	4	8.66
	L	-60	-46	14	8.09
	L	-48	-24	2	7.69
	L	-66	-26	12	6.46
	L	-62	-30	10	6.24
	L	-64	-18	4	5.55
	L	-48	0	-12	4.80
Inferior occipital gyrus	R	32	-80	-10	9.06
	L	-32	-72	-8	9.06
	R	34	-82	-6	8.94
	L	-34	-78	-4	7.86
	L	-32	-86	-4	7.29
Middle frontal gyrus	R	42	32	30	8.70
	L	-46	34	30	5.85
	L	-24	44	28	5.68
	L	-34	50	30	5.64
	L	-38	46	34	4.44
Inferior parietal lobule	R	32	-50	42	8.46
	R	48	-42	46	8.11
Supramarginal gyrus	R	56	-40	30	8.44
	L	-56	-46	30	5.27
Fusiform	L	-34	-62	-12	8.28

	L	-36	-60	-14	8.21
SMA	R	14	12	58	8.21
	L	-8	16	46	7.89
	R	6	20	50	7.43
	L	-10	8	58	4.23
	L	-12	8	62	4.16
Precentral gyrus	L	-40	10	32	7.06
Inferior frontal gyrus (p. triangularis)	L	-46	18	8	6.84
	L	-44	26	28	5.61
Inferior frontal gyrus (p. orbitalis)	L	-48	18	-4	6.09
Middle occipital gyrus	L	-26	-92	6	5.97
	L	-20	-96	4	5.80
Precentral gyrus	L	-34	0	44	5.42
	L	-40	2	54	3.89
Precuneus	R	12	-74	56	5.32
	R	10	-64	50	4.68
	R	10	-68	56	4.48
(D) CONTROLS>PD-PLACEBO ("disease effect"), FDRc p<0.05					
Insula	R	44	20	0	5.34
Temporal pole	L	-52	14	-2	4.97
Subcortex (no label in Anatomy toolbox)	L	-6	-22	-8	4.78
	L	-2	-10	-6	4.13
	L	-4	-12	-4	4.10
Thalamus	R	14	-18	10	4.49
Middle frontal gyrus	R	26	56	10	4.41
	R	34	52	6	3.83
Inferior frontal gyrus (p. triangularis)	L	-40	46	4	4.38
Middle orbital gyrus	R	36	48	-4	4.34
Caudate nucleus	L	-12	2	12	4.31

	R	12	2	12	4.27
Middle cingulate cortex	L	-6	-2	44	4.20
Putamen	R	28	10	8	4.13
	R	30	8	-6	3.85
	R	30	2	-8	3.66
Inferior frontal gyrus (p. opercularis)	R	54	20	26	4.10
	R	54	12	28	4.05
	L	-46	10	2	4.00
	L	-60	8	8	3.60
Rolandic operculum	R	52	10	2	3.95
	R	60	12	0	3.53
	L	-60	10	4	3.42
SMA	L	-4	0	56	3.88
(E) PD-PLACEBO>PD-ATOMOXETINE ("drug effect"), p<0.001 uncorrected, min 50 voxels					
Caudate nucleus/putamen (no label in Anatomy toolbox)	L	-14	8	7	5.17
	L	-18	10	13	4.77
	L	-20	16	5	3.89
Fusiform gyrus	R	40	-60	-17	4.92
Cerebellum	R	42	-66	-19	4.52
Middle frontal gyrus	R	34	46	7	4.50

Supplementary Table 3.

Difference in sum log-model evidence after fixed effects Bayesian Model Selection given between the numerically most likely model in each group and next most likely model (Supplementary Figure 1). Variance across subjects of difference in log-model evidence given as the standard deviation.

**Group**	**Numerically most likely model (FFX BMS)**	**Numerically second most likely model (FFX BMS)**	**ΔSum F (FFX)**	**Mean ΔF per subject**	**Standard deviation ΔF across subjects**
Controls	NCp	NBp	29.58	1.48	7.08
PD-placebo	NAp	NBp	4.11	0.22	0.32
PD-atomoxetine	NCp	NAp	6.98	0.37	6.04

Supplementary Figure 1.

A fixed effects Model Selection provided the difference in log-model evidence between models, and the posterior model probabilities. See Table 2 (main manuscript) for difference in log-model evidence between most likely model in each group and next most likely model. The most likely model for each group is indicated by the differences in log-model evidence (ΔF, left panels), and the posterior model probabilities that are close to 1 for each group (middle panels). The random effects Model Selection provided the exceedance probability, and expected probability (right panels). Labels on the x-axis indicate the identity of the 20 models: L=linear, N=nonlinear, A to D=model families. Given that the exceedance probability does not exceed 0.9, a hierarchical model selection and Bayesian Model Averaging was also performed (see main text).

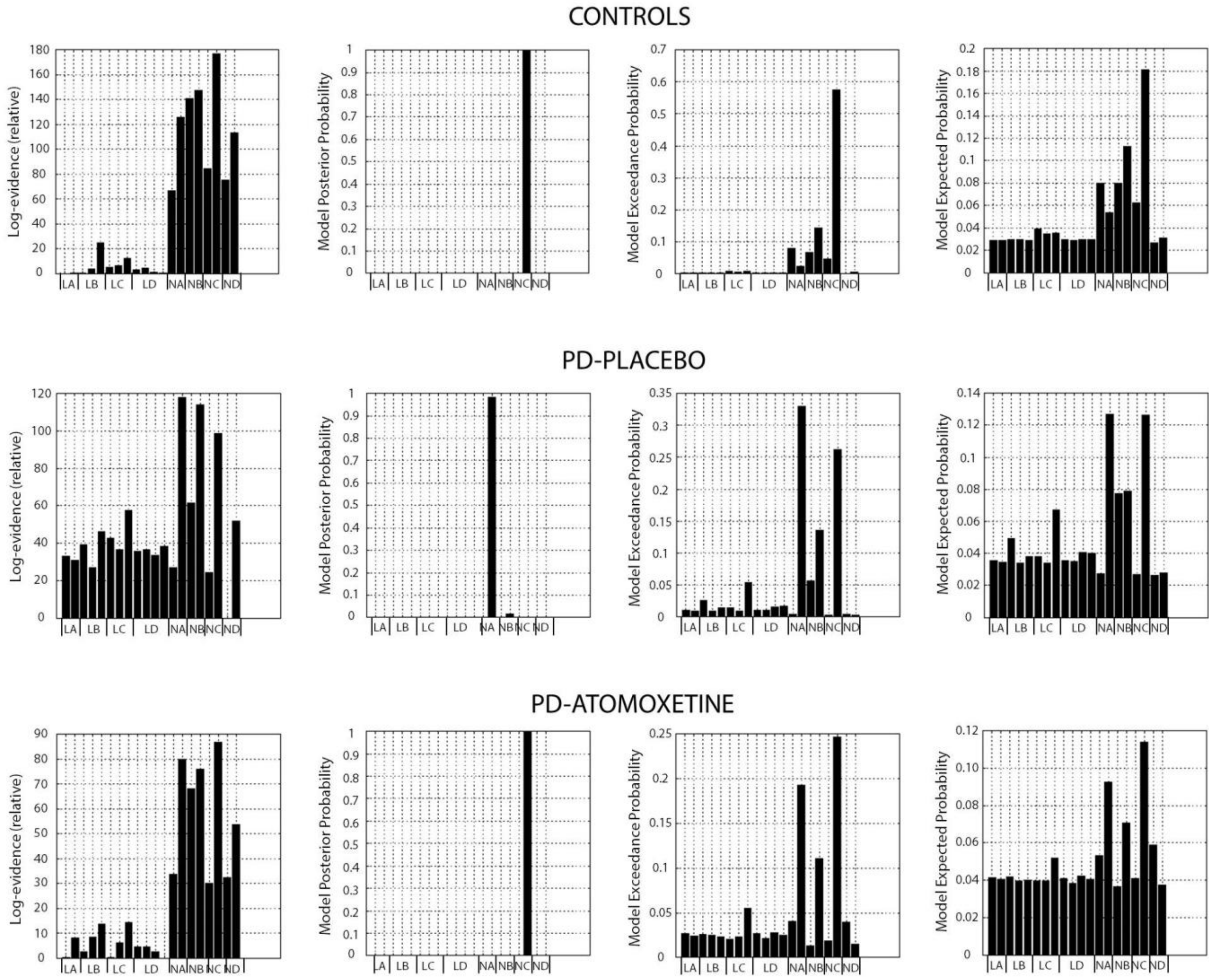

Supplement: Supplementary Data [file aww138_supplementary_data.zip › brain-2015-01535-File002.pdf]
